# Effect of Combined Kinematic Chain Exercise on Physical Function, Balance Ability, and Gait in Patients with Total Knee Arthroplasty: A Single-Blind Randomized Controlled Trial

**DOI:** 10.3390/ijerph20043524

**Published:** 2023-02-16

**Authors:** Jungae An, Young-Wan Son, Byoung-Hee Lee

**Affiliations:** 1 Graduate School of Physical Therapy, Sahmyook University, Seoul 01795, Republic of Korea; 2 Department of Physical Therapy, Sahmyook University, Seoul 01795, Republic of Korea

**Keywords:** knee, exercise, gait, pain, total knee arthroplasty

## Abstract

Total knee arthroplasty (TKA) is an effective treatment for end-stage osteoarthritis. However, evidence of combined kinematic chain exercise (CCE) in early-phase rehabilitation after TKA remains lacking. This study investigated the effects of CCE training on physical function, balance ability, and gait in 40 patients who underwent TKA. Participants were randomly assigned to the CCE (*n* = 20) and open kinematic chain exercise (OKCE) groups (*n* = 20). The CCE and OKCE groups were trained five times a week (for 4 weeks) for 30 min per session. Physical function, range of motion (ROM), balance, and gait were assessed before and after the intervention. The time × group interaction effects and time effect as measured with the Western Ontario and McMaster Universities Osteoarthritis Index, ROM, Knee Outcome Survey-Activities of Daily Living, balancing ability (e.g., confidence ellipse area, path length, and average speed), and gait parameters (e.g., timed up-and-go test, gait speed, cadence, step length, and stride length) were statistically significant (*p* < 0.05). In the group comparison of pre- and postintervention measurements for all variables, the CCE group showed substantial improvements compared to the OKCE group (*p* < 0.05). Both groups showed significant within-group improvement from baseline to postintervention. Our results suggest that CCE training positively affects physical function, balance ability, and gait as an early intervention for patients undergoing TKA.

## 1. Introduction

Total knee arthroplasty (TKA) is performed to reduce pain and improve physical function and quality of life when daily life is threatened by severe osteoarthritis [1,2]. Several innovations in orthopedic and rehabilitation practice were introduced in the last few years to improve clinical outcomes after TKA [3,4,5,6,7].

Both primary and revised TKA procedures are expected to increase globally with the increasing population of older adults [8]. Indeed, in Korea, the number more than doubled over a 10-year period, increasing from 29,653 in 2007 to 63,253 in 2017 [9]. However, 1 year after TKA, walking speed decreased by 18%, stair climbing speed decreased by 51%, and quadricep muscle strength reduced by 40% compared to the older adults without TKA, and patients with TKA reported greater difficulty performing functional movements such as squatting, sideways movement, and turning [10]. Defects in the functional activity of patients with TKA involve knee joint range of motion (ROM), muscle weakness, balance and motor control ability, proprioceptive sensory degradation, and gait ability [11,12,13].

Currently, rehabilitation exercises for patients who have undergone TKA include balance, proprioceptive sensory increase, preoperative open-kinetic chain (OKCE), and close-kinetic chain exercises (CKCE), which help improve strength, physical function, balance, and walking ability [14,15,16,17]. Generally, muscle-strengthening exercises for the knee joint include CKCE and OKCE. Since the concept of the kinematic chain was first established, CKCE has attracted increasing attention as an effective rehabilitation technique for improving lower extremity function [18]. CKCE is a functional exercise that involves general movement by fixing the distal part of the limb and mutually contracting the muscles of the compound joint with stability [19]. Additionally, by performing polyarthric movements, the muscles are co-contracted such that they affect the pressure of the articular capsule and activate the mechanoreceptor; thus, they functionally implement sports activities and daily life movements, such as sitting and standing, step-up and step-down, and heel-off the floor [20,21,22]. In OKCE, the distal part of the limb moves freely while the proximal limb is fixed, and it is more effective for concentric contractions of the individual muscles. As a general rehabilitation method, OKCE is applied as an exercise method using an increase in ROM, manual resistance, and exercise with equipment [23]. 

In early-phase rehabilitation after TKA, postoperative exercise generally involves OKCE, including continuous passive motion (CPM), which improves strength and ROM and can provide a minimal load to the operated joint [24]. Although OKCE focuses on training by separating a joint or individual muscle group, these movements are different to those used in daily life, and the shearing force for a new joint could be increased [25,26]. To improve this outcome, the combination of CKCE and OKCE is considered for early-phase patients after TKA. Combined kinematic chain exercise (CCE) is a combination of CKCE and OKCE with the advantages of each kinetic chain exercise. Previous studies have suggested that CCE effectively shortens the existing hospitalization period, enables a smooth return to daily life [23,25,27], and improves the stability, muscle strength, physical function, and balance ability required for daily life after knee rehabilitation [20,28,29].

Despite the advantages of CCE in knee rehabilitation, limited studies have evaluated the performance of applying CCE to early-phase patients after TKA, and studies verifying the effects of CCE on physical function, balance ability, and gait are insufficient [28]. Therefore, we sought to determine whether CCE affects physical function, balance ability, and gait in early-phase patients after TKA, with the aim to guide the clinical implementation of CCK for the rehabilitation of TKA. 

## 2. Materials and Methods

### 2.1. Participants

**Figure 1:**
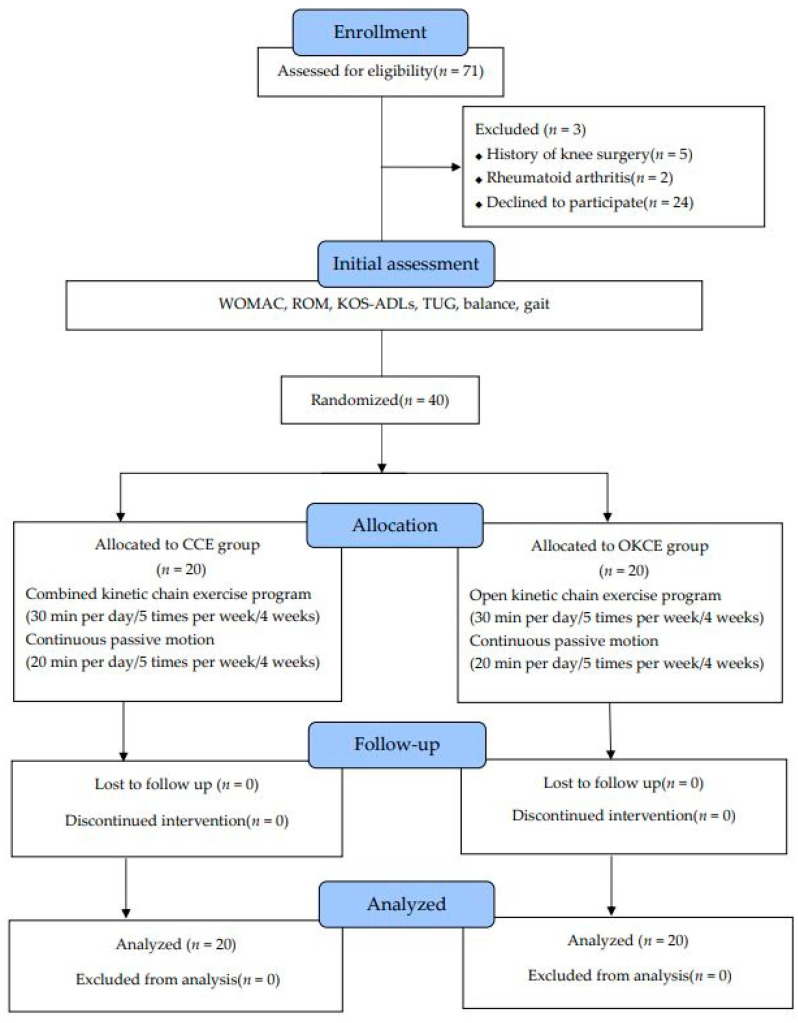
Flow diagram of the total experiment. CCE: combined kinetic chain exercise; OKCE: open kinetic chain exercise; WOMAC: the Western Ontario and McMaster Universities Osteoarthritis Index; ROM: knee range of motion; KOD-ADLs: Knee Outcome Survey-Activities of Daily Living.

This study enrolled 40 women who underwent TKA at an orthopedic surgery hospital. The inclusion criteria were as follows: older patients aged >65 years who could communicate with medical staff, those who underwent TKA owing to degenerative arthritis, and those with cemented TKA. The exclusion criteria were as follows: older patients aged >75 years; those with a history of knee joint surgery before TKA; those who possibly had a systematic inflammatory disease such as rheumatoid arthritis; those who had difficulty walking independently; those who had psychological problems or nervous disease; those who had previously received surgery for spine disease (Figure 1).

### 2.2. Ethical Statement

The purpose and requirements of this study were explained to the participants in detail. Patients could withdraw their consent to participate in the study at any time during the experimental procedure. Written informed consent was obtained from all patients. This study was approved by the Sahmyook University Institutional Review Board (approval number: 2-7001793-AB-N-012019064HR) and the Clinical Research Information Service (KCT0005823). Participants’ rights were protected according to the ethical principles of the Declaration of Helsinki. 

### 2.3. Study Design

This study had a single-blind, randomized controlled trial design. The assessors and participants were blinded to the grouping of each participant. Five physical therapists with more than 5 years of musculoskeletal field experience participated in the study procedures. The assessors were trained on equipment usage and measurement methods for 1 h twice a week before evaluating the participants. All evaluations and exercise training were conducted individually. G*Power Version 3.1.9.7 (Franz Faul, University Kiel, Germany, 2020) was used to calculate the sample size (two-tailed, effect value: 0.5, significant level α: 0.05, and power: 0.8) of the study. The number of required samples was 34. Considering a drop-out rate of 10–15%, 40 patients were recruited, and all study participants were pretested. To minimize the error related to the experiment, 40 patients were randomly assigned to the CCE and OKCE groups using the Research Randomizer program (http://www.randomizer.org/, accessed on 30 August 2019). 

### 2.4. Study Procedure

The experimental setting was an in-patient acute care unit at an orthopedic surgery hospital. All participants underwent TKA using a minimally invasive quadriceps-sparing technique with a high-flexion mobile prosthesis (Implantcast; GMBH Lüneburger Schanze, Buxtehude, Germany). From the 3rd day after TKA, the CCE and OKCE groups performed the exercise five times a week for 30 min (20 times for 4 weeks). Additionally, CPM was applied on the 1st day after TKA in both groups (once a day for 20 min, five times a week for 4 weeks). The CCE and OKCE groups were subjected to the same treatment conditions and environment. To examine the effect of the exercise program after the experiment, each group was administered the same procedure as the pretest and posttest. The baseline assessment was performed 1 day before the TKA procedure, and the posttest was performed 6 weeks after TKA. The Western Ontario and McMaster universities osteoarthritis (WOMAC), Knee Outcome Survey-Activities of Daily Living (KOS-ADLs), and an electronic goniometer were used to evaluate physical functions, and a Zebris PDM multifunction force measuring plate was used to measure balance ability and gait. The timed up-and-go (TUG) test was performed for dynamic balance ability.

### 2.5. Intervention

The CCK and CKCE groups performed each kinetic chain exercise starting on the 3rd day after TKA, and the participants each wore a knee brace and used a walker. The exercise program was based on that outlined in a previous study and was performed each week, considering each patient’s level of performance.

#### 2.5.1. Combined Kinetic Chain Exercise Program

In the 1st week of training, two sets of 10 repetitions (reps) were performed: quadriceps setting in the supine position, ankle full exercise in the supine position, wall slide, getting up and down from a chair, and standing up and off heels. For the 2nd to 3rd week of training, two sets of 10 reps were performed, including quadriceps setting while sitting, getting up and down from a chair, standing up and off heels, stepping up and down from a step box forward and sideward, and wall squatting. In the 4th week of training, getting up and down from a chair, stepping up and down from a step box forward and sidewards, hip abduction exercise while standing, and mini squats with a walker were performed in two sets of 10 reps for each movement, and two sets of one rep of toe gait were performed for 1 min [25,30,31]; the whole routine was performed for 30 min with a rest period of 30–40 s between sets (Table 1).

#### 2.5.2. Open Kinetic Chain Exercise Program 

In the 1st to 2nd week, the training program consisted of knee ROM exercises, quadriceps setting exercises by placing a foam roller under the knee in a supine position, straight leg raising, and ankle pull exercises in the supine position. In the 3rd to 4th weeks of training, ROM exercise of the knee, quadriceps setting in a sitting position, and hip abduction exercises while standing up were performed [32,33]; the rest period between the sets was 30–40 s for 30 min of exercise (Table 2).

### 2.6. Outcome Measurements 

#### 2.6.1. Physical Function

The WOMAC, ROM, and KOS-ADLs were used to measure the physical function in this study.

The WOMAC, which is widely used in patients with knee arthritis (intraclass correlation coefficient, ICC = 0.92), consists of 24 subjective questions about knee joint pain and functional limitations that can be felt daily [34,35]. The evaluation consisted of three subscales: pain, stiffness, and body function. Therefore, compared to other evaluation tools, the questions were formed on a simple and detailed scale such that knee joint pain and functional impairment can be evaluated very effectively [35]. In this study, the items on the pain scale ranged from 0–20 points, those on the stiffness scale ranged from 0–8 points, and those on the physical function scale ranged from 0–68 points. The three items were summed from 0–96, with a score of 0 indicating “none,” 4 indicating “very severe,” and a higher score indicating pain, stiffness, ROM, low level of body function, or physical disability.

Goniometer Biometrics (USA, 2008) was used to measure the flexion ROM of the knee joint (ICC = 0.99) [36]. To reduce the error between the measurements, the angle was adjusted to 0° for each measurement. The active joint ROM was measured thrice, and the average value was calculated. The examiner’s gaze was placed horizontally on the treatment table, and measurement was performed in a sitting position. The measurement position was fixed by placing the axis of the biometric goniometer at the lateral condyle of the tibia and the stabilization arm in the middle of the femur at the greater trochanter of the femur. The movement arm was measured by placing it in a straight line from the head of the calf to the lateral malleolus of the ankle.

The KOS-ADL was used to evaluate knee joint function in daily life. The symptoms included six items of influence on general daily living ability. The knee joint condition has 8 (of the total 14) items that influence the ability to perform functionally specific tasks and represents a reliable scale questionnaire to identify knee joint condition and functional ability for daily living (ICC = 0.97) [37].

#### 2.6.2. Balance

Balance ability was measured using a Zebris PDM platform (FDM 1.5, Zebris Medical GmbH, Isny, Germany, 2016), and “Zebris Win FDM” software was used for data collection and analysis. The Zebris PDM multifunction force-measuring plate consisted of a pressure mat (1580 mm in length × 605 mm in width × 21 mm in height; 11,264 sensors per 64 × 176 cm; range 1–120 N/cm^2^). The force measuring plate comprised four force sensors per 1 cm^2^, which measured postural sway during the patient’s standing posture and gait parameters while walking. The static and dynamic sampling pressure extraction rates were 2–5 Hz and 90 Hz, respectively, and the accuracy was within ±5%. The patient was asked to stand on the force plate and gaze at a fixed point, and postural sway, including the center of pressure 95% confidence ellipse area (CEA), the center of pressure path length (CPL), and the center of pressure and average velocity (CAV), was measured. The measurements were performed three times, and the average values were used for the analysis. The equipment showed high reliability (ICC = 0.8–0.9) [38].

#### 2.6.3. Gait 

Gait parameters, such as gait velocity, cadence, step length, and stride length, were measured using the Zebris PDM platform. The TUG test was used to measure functional mobility [39], balance, and walking ability. The patients were asked to get up from a sitting position on a chair with an armrest at a height of 46 cm at the same time as the examiner’s signal, make a 3 m round-trip return, and sit down; this was repeated thrice, and the average value was calculated [40]. The TUG test (ICC = 0.91–0.92) [41] showed high reliability.

### 2.7. Statistical Analysis

All tasks and statistics used in the analysis were based on SPSS ver. 22.0, which was used to calculate the means and standard deviations. All participants were normally distributed using the Shapiro–Wilk normality test. Descriptive statistics were used for the general characteristics of the participants. An independent sample *t*-test was performed to confirm the differences between the groups. A paired-sample *t*-test was performed to compare the differences before and after the intervention. The interaction effect between groups over time was analyzed using a two-way repeated measures analysis of variance (ANOVA). All statistical significance levels for the data were set to 0.05.

## 3. Results

A total of 71 patients were assessed for eligibility, and among them, 40 participants were randomized to complete the study intervention. All participants were women, and all had Kellgren–Lawrence grade 3–4 osteoarthritis before TKA. The demographic characteristics of the 40 patients who underwent TKA at baseline are shown in Table 3. 

### 3.1. Primary Outcomes 

The physical function values are listed in Table 4. The time × group interaction effect for all physical function values showed statistically significant interaction effects (*p* < 0.05) and a significant effect of time. The CCE group showed significant improvements in mean values compared to that of the OKCE group after the intervention (*p* < 0.05) (Table 4). 

### 3.2. Secondary Outcome

The balance ability metrics, CEA, CPL, and CAV, were measured using a force plate and are shown in Table 4. The results show a significant time × group interaction effect and time effect for the balance values (*p* < 0.05). The mean values of CEA, CPL, and CAV significantly improved in the CCE group compared to those of the OKCE group after the intervention (*p* < 0.05), and significant differences were observed between the groups (*p* < 0.05) (Table 5).

Table 5 shows the gait velocity, cadence, step length, and stride length from gait analysis and the TUG time of the participants. A statistically significant group factor effect on CEA levels was observed (*p* = 0.08). A time × group interaction effect (*p* < 0.05) and time effect (*p* < 0.05) were observed for all gait parameters. The CCE group performed significantly better in gait parameters after the intervention than the OKCE group (*p* < 0.05) (Table 6).

## 4. Discussion

This study found that the 4-week CCE program improved physical function, balance ability, and gait in early-phase patients after TKA.

### 4.1. Effects of Physical Function

TKA relieves pain and improves physical function while decreasing functional movement abilities, such as climbing up and down stairs and walking. After surgery, the patients’ quadriceps femoris and hamstrings are weakened, which leads to the occurrence of muscular atrophy and neuromuscular defects [42,43,44]. Postoperative rehabilitation is performed to improve performance during the functional activities that occur in daily life, and various rehabilitation methods must be implemented to recover functional exercise performance, muscle strength, balance ability, and proprioception [45]

Olagbegi OM et al. [31] applied CCE to patients with osteoarthritis for a 12-week period. The results showed significant differences in average daily pain, pain before walking, and pain after walking (*p* < 0.05). CCE training can more effectively reduce pain and functional limitations in patients. Similar results were obtained in the current study, in which the WOMAC, a tool for evaluating a physical function, showed significant improvements (*p* < 0.05). The pain, stiffness, and physical function scores in the CCE group decreased from 12.60 preoperatively to 3.85 points postintervention, 4.95 points to 1.50 points, and 48.60 points to 24.20 points, respectively. MacKay et al. [46], in their systematic review, suggested a minimal clinically important difference (MCID) for WOMAC pain (13.3–36) and WOMAC function (1.8–33), as well as the patient-acceptable symptom state cut-off for WOMAC pain (25–28) and WOMAC function (32.3–36) for 6 to 12 months after TKA. Although we could not achieve the suggested MCID, our results revealed score improvement only 6 weeks after TKA. The CCE group showed a greater improvement in ROM than the OKCE group, which was from 108.02° to 137.92° (*p* < 0.01). CCE consisted of sitting and getting up from a chair, wall squats, mini squats with a walker, and walking with heels. CCE enhances the proprioceptive sensory muscles, ligaments, and tendons damaged after surgery and increases the stability of the knee, hip, and ankle joints [21] to increase the range of body movement and facilitate participation in the appropriate physical activity. Improving muscle tone, circulatory function, and flexibility [44] is thought to affect the knee disability index on the scales of muscle stiffness and pain, ROM, and function. In the current study, the KOS-ADL score increased from 24.80 points at baseline to 46.10 points after intervention in the CCE group (*p* < 0.001). A significant difference was also found in the group comparison (*p* < 0.05). Mizner and colleagues [43] found that the evaluation scores for walking, getting up from a chair, and going up and down the stairs increased compared to the preoperative evaluation score 1 month after TKA. In the functional part of the KOS-ADL, except for squatting and kneeling, all items increased in the 1 to 12 months after surgery. Significant differences were found between knee joint flexion and extension ROM and KOS-ADL scores (*p* < 0.05). CCE training prevents co-contraction of the quadriceps femoris and increases the muscle activity of the lower extremities through strength training to improve eccentric contraction similar to the repeated movements of daily life; better scores of the KOS-ADL indicate improved stiffness and knee movement [47].

### 4.2. Effects of Balance 

The patients who underwent TKA complained of discomfort due to incomplete bodily dysfunction. Muscle strengthening exercises, such as kinetic chain exercises, can improve balance by boosting the strength and thickness of the knee extensor muscles (*p* < 0.05) [26]. Regarding the balance ability of the CCE group, their CEA, CPL, and CAV measurements decreased from 456.42 mm^2^ to 285.30 mm^2^, from 130.20 mm to 99.29 mm, and from 14.49 mm/s to 10.24 mm/s, respectively (*p* < 0.05), confirming that the CCE program improved balance ability. Kwon et al. [23] applied CKCE training to 33 healthy adults and demonstrated that the front and rear sway distances, the inner and outer sway distances, and the total sway distance were reduced from 748.2 mm to 641.6 mm, from 635.1 mm to 508.9 mm, and from 1093.3 mm to 914.9 mm in the experimental group (*p* < 0.05), respectively. Additionally, Lim et al. [48] demonstrated that CKCE and OKCE training improved knee joint position sensory input and affected the patient’s balance ability. The CCE training program can activate the functions of vestibular, somatic, and visual sensory, and it can maintain human balance ability [49]. A person’s static balance can be improved [49], and activation of the initial quadriceps muscle can be increased by mutual contraction of the knee extensor and flexor muscles through exercises such as sitting and standing, wall squats, and mini squats with a walker [20].

### 4.3. Effects of Gait 

After TKA, the patients experienced a decreased frequency of step length owing to slow gait speed and a decreased knee joint flexion angle during the stance and swing phases. Smith et al. [50] reported that gait dysfunction (due to an abnormal gait pattern) in some patients persisted for 12 to 18 months after TKA. The minimum speed required for walking in daily life is 0.42 m/s (0.4–0.8 m/s with some limitations); the speed required for independent walking is 0.8 m/s or more [51,52]. In this study, the walking speed was significantly improved (from 2.32 km/h (0.64 m/s) to 2.68 km/h (0.74 m/s)) within the CCE group (*p* < 0.001), and there was a significant difference in the CCE group compared to the OKCE group (*p* < 0.01). Cadence was significantly improved (from 98.49 steps/min to 107.68 steps/min) in the CCE group (*p* < 0.001). A significant difference in cadence was found between the CCE and OKCE groups (*p* < 0.01). The TUG time decreased significantly from 13.92 s to 10.47 s in the CCE group (*p* < 0.001), and a significant difference of TUG time was found in the CCE group compared to the OKCE group (*p* < 0.01). The phase related to gait speed in the gait cycle is between the midstance and terminal stance phases, with the gluteus maximus and calf muscles being the main acting muscles [53]. The mini-squat, sit-to-stand, and wall squat exercises of the CCE in this study strengthened the lateral rotation muscles of the hip joint, such as the gluteus maximus on the weak side [51,52]. The torque at the subtalar joint (the internal torque) and the torque at the metatarsophalangeal joint interact because of the heel-off and toe-gait motions, which improved the action of the calf muscle in the terminal stance phase and enhanced the driving force for gait [54,55]. Additionally, proprioceptive sensation and stability around the knee joint were improved using polyarticular muscles in a weight-supported state [56].

Ouellet and Moffet [57] reported that stride length was 90 cm in 2 months after TKA and 140 cm in healthy participants, whereas the ratios of the stance phase were 68% in 2 months after TKA and 65% for healthy participants during gait (*p* < 0.05). This result can be explained by the fact that as double-support time increases, single-support time for the weak-side leg decreases. Winter et al. [58] reported that the prolonged double-support time of the weak-side leg was a systematic strategy to facilitate safer walking to support the weight of the weak-side leg. When walking, step length or stride represents the propulsive force of the leg, and the patient is limited in the ability to shift the body center to the weak-side leg. Therefore, a short stride length and low cadence are due to decreased balance ability. A slow gait appears in hemiplegic patients, owing to a decrease in step length. This change is a direct cause of the decrease in balance ability during gait, whereas an increase in stride length may result in high-speed gait [59]. In this study, the step length during walking increased significantly from 37.38 cm to 39.89 cm in the CCE group (*p* < 0.001). A significant difference in the step length was observed between the CCE and OKCE groups (*p* < 0.01). Stride length increased significantly from 77.99 cm to 84.19 cm in the CCE group (*p* < 0.001). A significant difference in stride length was observed between the CCE and OKCE groups (*p* < 0.01). This result can be explained by the fact that CCE training performs polyarticular movements rather than monoarticular movements compared to OKCE training alone; therefore, muscle strength is boosted in various muscle groups, and activity is increased by enhancing motor unit mobilization to increase the stability of lower extremity joints [60]. The increased strength of the quadriceps femoris and eccentric contraction of the hamstrings may improve the contact force between the foot and the floor [61]. Additionally, the swing phase affects the step length and stride length in gait, and the terminal swing phase, in which the lower limb is extended forward [62], is the main phase in the gait stage. Step and stride lengths could be sufficiently improved through OKCE. Nevertheless, the reason for further improvement is that the ability to move the center of the body forward is improved by increasing the stability of the transition from the middle stance phase of the weak-side lower limb to the terminal stance phase. Additionally, the step length and stride length may increase with improvement in the terminal swing phase. 

This study had several limitations. We evaluated the short-term effect of CCE after 4 weeks of intervention. However, the long-term effects could not be determined owing to a lack of follow-up. Moreover, as the participants (only women) were recruited from specific countries and regions, the results cannot represent all patients who underwent TKA. Additionally, we could not control the drugs and injections used for pain control in the in-patient unit. Therefore, in a future study, considering these limitations, a long-term exercise plan should be implemented for all patients who underwent TKA, and it should be confirmed whether the impact of CCE is effective throughout the follow-up. Additional studies are needed to provide evidence to support CCE training in patients who underwent TKA.

## 5. Conclusions

Our results reveal that CCE training improved physical function, balance, and gait in women in the early phase after TKA. Therefore, CCE training can serve as a basis for functional gait enhancement; thus, it improves the standard of living of patients with TKA (daily living, preventing falls, and reducing the fear of walking). We suggest that CCE training as an early-phase intervention is effective for patients after TKA.

## Figures and Tables

**Table 1:** Combined kinetic chain exercise program.

Week	Contents of Program	Times
1st week	Quadriceps setting in the supine position Ankle full exercise in a supine Wall slide Getting up and down from a chair Standing up and off heels	10 times, 2 sets
2nd to 3rd week	Quadriceps setting while sitting Getting up and down from a chair Standing up and off heels Wall squat Stepping up and down the step box forward and sideward	10 times, 2 sets
4th week	Getting up and down from a chair Hip abduction exercise while standing Stepping up and down from a step box forward and sideward Mini squat with a walker Toe gait	10 times, 2 sets
Total exercise time was 30 min with a rest time of 30–40 s between sets.		

**Table 2:** Open kinetic chain exercise program.

Week	Open-Kinetic Chain Exercise Program	Reps
1st to 2nd week	Knee range of motion exercise Quadriceps setting in a supine position with a foam roller under the knees Straight leg-raising Ankle full exercise in a supine position	Each 5 s 10 reps/6 sets
3rd to 4th week	Knee range of motion exercise Ankle full exercise in a supine position Quadriceps setting in a sitting position Hip abduction exercise in a standing position	Each 5 s 10 reps/6 sets
Total exercise time was 30 min with a rest time of 30–40 s between sets.		

**Table 3:** Demographic characteristics (*n* = 40).

Characteristics	CCE Group (*n* = 20)	OKCE Group (*n* = 20)	*X*^2^/F(*p)*
Surgery side (left/right)	10/10	9/11	0.309 (0.759)
Age (years)	71.60 ± 3.53	70.04 ± 2.47	1.244 (0.221)
Height (cm)	152.47 ± 6.05	151.34 ± 5.82	0.599 (0.605)
Weight (kg)	61.08 ± 8.67	59.57 ± 9.54	0.522 (0.553)
BMI (kg/m^2^)	26.15 ± 2.89	25.92 ± 3.30	0.237 (0.814)

CCE: combined kinetic chain exercise; OKCE: open kinetic chain exercise; BMI: body mass index. Values are expressed as the mean ± standard deviation. Significant: *p* < 0.05.

**Table 4:** Comparisons of physical function.

Variable		CCE Group (*n* = 20)	OKCE Group (*n* = 20)	t (*p*)	Time	Group	Time × Group
					*F* (*p*)	*F* (*p*)	*F* (*p*)
WOMAC (Pain)	Pretest	12.60 ± 1.66	11.85 ± 2.05	1.266 (0.213)	304.42 (0.000)	6.060 (0.018)	26.725 (0.000)
	Posttest	3.85 ± 1.66	7.10 ± 2.55				
	Mean difference	8.75 ± 2.51	4.75 ± 2.38	5.170 (0.000)			
	95% CI	(7.575 to 9.925)	(3.653 to 5.865)				
	t (*p*)	15.587 (0.000)	8.920 (0.001)				
WOMAC (Stiffness)	Pretest	4.95 ± 1.05	4.40 ± 0.88	1.793 (0.081)			
	Posttest	1.50 ± 0.68	2.65 ± 0.81				
	Mean difference	3.45 ± 1.35	1.75 ± 1.11	4.325 (0.000)	175.04 (0.000)	2.447 (0.126)	18.709 (0.000)
	95% CI	(2.815 to 4.085)	(1.227 to 2.273)				
	t (*p*)	11.376 (0.000)	7.000 (0.000)				
WOMAC (Physical function)	Pretest	48.60 ± 6.93	47.70 ± 9.02	0.354 (0.725)			
	Posttest	24.20 ± 5.46	31.25 ± 7.03				
	Mean difference	24.40 ± 8.48	16.45 ± 7.02	3.227 (0.003)	275.01 (0.000)	2.555 (0.118)	10.416 (0.003)
	95% CI	(20.49 to 28.38)	(13.16 to 19.74)				
	t (*p*)	12.856 (0.000)	10.476 (0.000)				
ROM	Pretest	108.02 ± 7.07	109.92 ± 9.90	–0.696 (0.491)			
	Posttest	137.92 ± 3.15	132.78 ± 6.70				
	Mean difference	29.90 ± 8.32	22.86 ± 7.47	2.815 (0.008)	444.98 (0.000)	0.751 (0.392)	7.923 (0.008)
	95% CI	(26.00 to 33.795)	(19.36 to 26.36)				
	t (*p*)	–16.065 (0.000)	–13.682 (0.000)				
KOS-ADLs	Pretest	24.80 ± 10.10	30.20 ± 9.42	–1.748 (0.089)			
	Posttest	46.10 ± 8.99	43.80 ± 8.50				
	Mean difference	21.30 ± 8.46	13.60 ± 8.81	2.817 (0.008)	163.03 (0.000)	0.357 (0.554)	7.936 (0.008)
	95% CI	(16.065 to 25.263)	(9.47 to 17.73)				
	t (*p*)	–11.250 (0.000)	–6.899 (0.000)				

CCE: combined kinetic chain exercise; OKCE: open kinetic chain exercise; WOMAC: the Western Ontario and McMaster Universities Osteoarthritis Index; ROM: knee range of motion; KOD-ADLs: Knee Outcome Survey-Activities of Daily Living. Values are expressed as the mean ± standard deviation. 95% CI: 95% confidence interval; significant: *p* < 0.05; F: two-way repeated-measure analysis of variance.

**Table 5:** Comparisons of balance parameters.

Variable		CCE Group (*n* = 20)	OKCE Group (*n* = 20)	t (*p*)	Time	Group	Time × Group
					*F* (*p*)	*F* (*p*)	*F* (*p*)
CEA (mm^2^)	Pretest	456.42 ± 188.43	517.72 ± 231.21	–0.919 (0.364)			
	Posttest	285.30 ± 212.74	403.15 ± 248.05				
	Mean difference	171.11 ± 76.02	114.57 ± 89.09	2.159 (0.037)	304.42 (0.000)	6.060 (0.018)	26.725 (0.000)
	95% CI	(135.54 to 206.70)	(72.88 to 156.27)				
	t (*p*)	10.066 (0.000)	5.751 (0.000)				
CPL (mm)	Pretest	130.20 ± 32.68	127.28 ± 38.69	0.258 (0.798)			
	Posttest	99.29 ± 29.95	109.22 ± 37.00				
	Mean difference	30.90 ± 15.30	18.05 ± 13.23	2.842 (0.007)	119.00 (0.000)	1.699 (0.200)	4.661 (0.037)
	95% CI	(23.75 to 38.07)	(11.86 to 24.25)				
	t (*p*)	9.031 (0.000)	6.102 (0.000)				
CAV (mm/sec)	Pretest	14.49 ± 3.12	14.56 ± 4.75	–0.053 (0.958)			
	Posttest	10.24 ± 3.26	11.75 ± 4.08				
	Mean difference	4.24 ± 1.32	2.80 ± 1.49	3.228 (0.003)	117.13 (0.000)	0.106 (0.746)	8.076 (0.007)
	95% CI	(3.63 to 4.87)	(2.11 to 3.51)				
	t (*p*)	3.228 (0.003)	8.407 (0.000)				

CCE: combined kinetic chain exercise; OKCE: open kinetic chain exercise; CEA: confidence ellipse area; CPL: center of pressure path length; CAV: center of pressure average velocity. Values are expressed as the mean ± standard deviation; 95% CI: 95% confidence interval; significant: *p* < 0.05; F: two-way repeated-measure analysis of variance.

**Table 6:** Comparisons of gait parameters.

Variable		CCE Group (*n* = 20)	OKCE Group (*n* = 20)	t (*p*)	Time	Group	Time × Group
					*F* (*p*)	*F* (*p*)	*F* (*p*)
TUG (s)	Pretest	13.92 ± 2.41	13.05 ± 1.76	1.299 (0.202)			
	Posttest	10.47 ± 1.45	11.19 ± 2.14				
	Mean difference	3.45 ± 1.41	1.86 ± 1.36	3.626 (0.001)	146.42 (0.000)	0.015 (0.903)	13.150 (0.001)
	95% CI	(2.79 to 4.12)	(1.22 to 2.50)				
	t (*p*)	10.916 (0.000)	6.108 (0.000)				
Velocity (km/h)	Pretest	2.32 ± 0.44	2.49 ± 0.63	–0.981 (0.333)			
	Posttest	2.68 ± 0.41	2.65 ± 0.61				
	Mean difference	0.35 ± 0.26	0.15 ± 0.24	2.526 (0.016)	249.99 (0.000)	0.430 (0.516)	10.421 (0.003)
	95% CI	(0.23 to 0.48)	(0.03 to 0.26)				
	t (*p*)	–5.998 (0.000)	–2.764 (0.013)				
Cadence (step/min)	Pretest	98.49 ± 7.47	102.18 ± 8.55	–1.450 (0.155)			
	Posttest	107.68 ± 6.99	104.81 ± 8.27				
	Mean difference	9.18 ± 4.15	2.63 ± 3.58	5.337 (0.000)	92.683 (0.000)	0.029 (0.886)	28.485 (0.000)
	95% CI	(7.24 to 11.13)	(0.96 to 4.30)				
	t (*p*)	–9.879 (0.000)	–3.285 (0.004)				
Step length (cm)	Pretest	37.38 ± 5.71	39.54 ± 4.85	–1.286 (0.206)			
	Posttest	39.89 ± 5.92	40.41 ± 4.57				
	Mean difference	2.49 ± 1.48	0.86 ± 1.24	3.766 (0.001)	49.146 (0.000)	1.060 (0.310)	9.533 (0.004)
	95% CI	(1.81 to 3.19)	(0.28 to 1.45)				
	t (*p*)	–7.509 (0.000)	–3.114 (0.006)				
Stride length (cm)	Pretest	77.99 ± 13.53	84.08 ± 11.78	–1.516 (0.138)			
	Posttest	84.19 ± 14.90	86.48 ± 11.53				
	Mean difference	6.19 ± 3.39	2.40 ± 4.31	3.088 (0.004)	60.206 (0.000)	0.657 (0.423)	14.183 (0.001)
	95% CI	(4.61 to 8.16)	(0.39 to 4.43)				
	t (*p*)	–8.155 (0.000)	–2.498 (0.022)				

CCE: combined kinetic chain exercise; OKCE: open kinetic chain exercise; TUG: timed up-and-go. Values are expressed as mean ± standard deviation; 95% CI: 95% confidence interval; significant: *p* < 0.05; F: two-way repeated-measure analysis of variance.

## Data Availability

Not applicable.

## Abbreviations

BMI: body mass index; CAV: center of pressure average velocity; CCE: combined kinetic chain exercise; CEA: confidence ellipse area; CKCE: close-kinetic chain exercises; CPL: center of pressure path length; CPM: continuous passive motion; KOD-ADLs: Knee Outcome Survey-Activities of Daily Living; MCID: minimal clinically important difference; OKCE: open kinetic chain exercise; ROM: range of motion; TKA: total knee arthroplasty; TUG: timed up-and-go; WOMAC: The Western Ontario and McMaster Universities Osteoarthritis Index.
